# *Streptococcus pneumoniae* detects and responds to foreign bacterial peptide fragments in its environment

**DOI:** 10.1098/rsob.130224

**Published:** 2014-04-09

**Authors:** Lucy J. Hathaway, Patrick Bättig, Sandra Reber, Jeannine U. Rotzetter, Suzanne Aebi, Christoph Hauser, Manfred Heller, Aras Kadioglu, Kathrin Mühlemann

**Affiliations:** 1 Institute for Infectious Diseases, University of Bern, Bern, Switzerland; 2 Department of Infectious Diseases, University Hospital, Bern, Switzerland; 3 Department of Clinical Research, University Hospital, Bern, Switzerland; 4 Department of Clinical Infection, Microbiology and Immunology, Institute of Infection and Global Health, University of Liverpool, Liverpool, UK

**Keywords:** *Streptococcus pneumoniae*, bacteria, peptide, interspecies communication, non-encapsulated

## Abstract

*Streptococcus pneumoniae* is an important cause of bacterial meningitis and pneumonia but usually colonizes the human nasopharynx harmlessly. As this niche is simultaneously populated by other bacterial species, we looked for a role and pathway of communication between pneumococci and other species. This paper shows that two proteins of non-encapsulated *S. pneumoniae*, AliB-like ORF 1 and ORF 2, bind specifically to peptides matching other species resulting in changes in the pneumococci. AliB-like ORF 1 binds specifically peptide SETTFGRDFN, matching 50S ribosomal subunit protein L4 of Enterobacteriaceae, and facilitates upregulation of competence for genetic transformation. AliB-like ORF 2 binds specifically peptides containing sequence FPPQS, matching proteins of *Prevotella* species common in healthy human nasopharyngeal microbiota. We found that AliB-like ORF 2 mediates the early phase of nasopharyngeal colonization *in vivo*. The ability of *S. pneumoniae* to bind and respond to peptides of other bacterial species occupying the same host niche may play a key role in adaptation to its environment and in interspecies communication. These findings reveal a completely new concept of pneumococcal interspecies communication which may have implications for communication between other bacterial species and for future interventional therapeutics.

## 2. Introduction

In nature, most bacteria live in multi-species communities known as microbiota, which when bound together by extracellular matrix are called biofilms. In such environments, interactions between bacterial species must be important but are mostly unknown. We know more about intraspecies communication via diffusible signal molecules called autoinducers [1]. Gram-positive bacteria use amino acids and peptides as signal molecules; Gram-negative bacteria use *N*-acyl-l-homoserine lactones (*N*-AHLs), which mediate quorum sensing leading, for example, to biofilm development [1]. So far only one cell–cell signalling system, autoinducer-2, is known to be shared by Gram-positive and Gram-negative bacteria. It has multiple effects including on virulence and has been proposed to act in interspecies communication [2].

In addition, diverse non-canonical d-amino acids are produced by several bacterial species during stationary phase of growth and modulate synthesis of peptidoglycan of the cell wall [3]. Release of d-amino acids from one species could affect other bacterial species in the same niche, for example by inducing biofilm disassembly [3,4]. In *Vibrio cholerae,* production of the d-amino acids seems to be a stress response enabling the bacteria to respond to environmental changes [5].

Antibiotics may also naturally function in interspecies signalling at subinhibitory concentrations [6]. Antibiotics elicit a stress response and induce competence for genetic transformation in *Streptococcus pneumoniae*, increasing the ability to take up foreign DNA [7]. Transformation is thought to contribute to genetic plasticity and therefore adaptation of *S. pneumoniae* to a hostile environment by acquiring new, potentially useful, genes [8].

*Streptococcus pneumoniae,* although a major human pathogen, usually lives harmlessly in the human nasopharynx alongside commensal streptococcal species and other commensal bacterial groups of the nasopharyngeal microbiota, including *Bacteroides, Fusobacterium*, Firmicutes, Actinobacteria and Proteobacteria [9]. Disturbance of the nasopharyngeal ecosystem can lead to abnormal upper-airway colonization, for example by the normally intestinal colonizers Enterobacteriaceae [10]. We hypothesized that *S. pneumoniae* and commensal streptococci must have a way to sense and respond to their surrounding microbiota and changes in its composition.

A subpopulation of pneumococci lack a polysaccharide capsule and these non-encapsulated strains are estimated to make up 0.5–2.2% of *S. pneumoniae* isolated from sterile sites and 10–15% of those isolated from the nasopharynx [11–13]. This may be an underestimate as the small colonies they produce on agar plates might be overlooked in diagnostic laboratories. We have shown previously that a subset of non-encapsulated *S. pneumoniae* carries two extra genes, *aliB*-like ORF 1 and ORF 2, in place of capsule genes [14]. We predict that the *aliB*-like ORFs encode substrate-binding proteins of an unknown ATP-binding cassette (ABC) transporter(s). ABC transporters are important in *S. pneumoniae* virulence [15]. They generally consist of two permease domains embedded in the membrane, two ATP-binding domains which provide the energy for transport and one or more substrate-binding domains. It is these substrate-binding domains that determine the specificity of the transporter and bring the substrate to the permease for uptake. The AliB-like ORFs are named for their sequence homology to AliB substrate-binding protein of the Ami-AliA/AliB permease, found in all *S. pneumoniae* strains, which is proposed to take up peptides to sense the nutritional environment [16].

Here, we investigated whether the AliB-like ORFs can bind and respond to peptides that may be secreted by bacterial neighbours and so play a role in interspecies communication and adaptation of *S. pneumoniae* to its environment.

## 3. Material and methods

### 3.1. Bacterial culture

Bacteria, stored at −80°C using Protect bacterial preservers (Technical Service Consultants, Heywood, UK), were grown on Columbia sheep blood agar (CSBA) plates at 37°C, 5% CO_2_. Overnight cultures were prepared with 3–10 colonies in 5 ml brain heart infusion (BHI) broth (Becton Dickinson and Company, le Pont de Claix, France) containing 5% fetal calf serum (FCS; Biochrom KG, Berlin, Germany).

### 3.2. Bacterial strains

For Swiss non-encapsulated nasopharyngeal pneumococcal isolate 110.58, MLST 344 [14], mutants were constructed to inactivate one or both *aliB*-like ORFs (see the electronic supplementary material, figure S1). Construction of the mutant ΔORF 1 + 2 was described previously [14]. Restriction fragment length polymorphism (RFLP)-typing confirmed transformants originate from strain 110.58 [17]. Southern blotting and PCR to determine the presence of the ORFs and transformation of *Escherichia coli* and *S. pneumoniae* were performed as described previously [14,18].

#### 3.2.1. Construction of mutant ΔORF 1

A 1381 bp fragment of *dexB* of strain 110.58 and chloramphenicol acetyltransferase (*cat*) gene from plasmid pJS3copG7 (kindly provided by M. Espinosa, Centro de Investigaciones Biologicas, Madrid, Spain [19]) were inserted into the multiple cloning site of plasmid pBluescript KSII (Stratagene). Downstream of *cat* a 1625 bp fragment of *aliB*-like ORF 1 (lacking the 5’ end) was inserted. Primers used were: dexB_f145_XbaI, dexB_b1504_BamHI, cat_Spn_F1_HindIII and cat_Spn_B1_HindIII [14]. To amplify the 3′-end of *aliB*-like ORF 1, we used primers 110.58_FI3.2_ClaI: CCATCGATGGGTATGCGAAAGTTAAAG CTC, and 104b832.13_XhoI: CCGCTCGAGTGAGCATTCGATTCCAGTTTT. The *dexB-cat-ORF1* construct was amplified using primers dexB_f145_XbaI and 104b832.13_XhoI and used to transform *S. pneumoniae* strain 110.58. Recombinant clones were selected on CSBA plates containing 3 mg l^−1^ chloramphenicol. PCR confirmed deletion of the 5′ end of *aliB*-like ORF 1 and real-time RT-PCR confirmed that deletion of *aliB*-like ORF 1 did not cause a polar effect by altering the expression level of *aliB*-like ORF 2.

#### 3.2.2. Construction of mutant ΔORF 2

A fragment of *aliB*-like ORF 2 of 110.58 was amplified by PCR (using primers 104.72_104FI3.6_222311: AGATGCCAAATGGTTCACGG, and 106_106b832.5_222506: CATCTTTGAGCATCTTAGTG) and inserted into pGEM-T Easy Vector (Promega, Wallisellen, Switzerland). A chloramphenicol cassette with an ami promoter [16] (primers: Pcat_F_NheI: CTAGCTAGCgaaaatttgtttgatttttaatggataatgtgatataatgGTTCAACAAACGAAAATTGGATAAAGT, and Pn_cat_B1_NheI: ctagctagcCGGGGCAGGTTAGTGACAT) was cloned into the *aliB*-like ORF2 fragment at the NheI restriction site. Strain 110.58 was transformed with the plasmid containing the construct. Recombinant mutants selected on CSBA plates containing 3 mg l^−1^ chloroamphenicol were analysed by PCR.

Laboratory *S. pneumoniae* strain R6, used as a control, was a kind gift from Philippe Moreillon (Centre Hospitalier Universitaire Vaudois, Lausanne, Switzerland).

### 3.3. PCR and real-time RT-PCR

To assess the presence of *aliB*-like ORFs the following primers were used: forward primer binding in *aliB*-like ORF 1 110_FI3.4 (5′-AACACTTGGAACGGAGA-3′), reverse primer binding in *aliB-like* ORF 1 104_b832.14 (5′-GCCCTTTGTTATACCTAGATGTTTC-3′), forward primer binding in *aliB*-like ORF 2 104_FI3.6 (5′-AGATGCCAAATGGTTCACGG-3′) and reverse primer binding in *aliB-*like ORF 2 104_b832.10 (5′-GAAATCTTTTAACAAATAAGGTCCG-3′). PCR was performed using FastStart Taq (Roche): 94°C for 5 min, then 94°C for 30 s, 50°C for 30 s, 72°C for 2 min for 30 cycles then 72°C for 10 min.

Gene expression was quantified as described previously [20] and normalized against 16S using the following primers and probes: *aliB*-like ORF 1 forward primer, 5′-GGTTCTGCCTTGAAACATCTAGGT-3′; reverse primer, 5′-CCAACTTTCGCCACAACTTCA-3′; probe, 6-carboxyfluorescein (6-FAM)-AAAGGGCAAGGATCC-minor groove binder (MGB); *aliB*-like ORF 2 forward primer, 5′-CTCTACAGCGGACCTTATTTGTTAAAAGA-3′; reverse primer, 5′-CGGATGGTCAATTCTTGGTCTGA-3′; probe, 6-FAM-CTTTGTCATGATCATAGTAATGC-MGB; 16S forward primer, 5′-GACGATACATAGCCGACCTGAGA-3′; reverse primer, 5′-GTAGGAGTCTGGGCCGTGTCT-3′; probe, 6-FAM-CCAGTGTGGCCGATC-MGB.

### 3.4. Expression and characterization of AliB-like ORF 1 and ORF 2 proteins

Proteins AliB-like ORF 1 and ORF 2 were expressed in *E. coli* Rosetta strain (Novagen, Darmstadt, Germany) and purified using an N-terminal glutathione S-transferase (GST)-tag (AliB-like ORF 1) or C-terminal His_6_-tag (AliB-like ORF 2). SDS-PAGE of the proteins before and after purification is shown in the electronic supplementary material, figure S2. For AliB-like ORF 1, forward primer 5′-TTTCCCGGGCGAAAACATTTTCTTATGTTTATG-3′ and reverse primer 5′-AAA CTCGAGTTAATGATGATGATGATGATGACCTCATTTTACGTGTTTTTCTAAA-3′ were used to amplify the full-length protein excluding the 5′ portion encoding the predicted N-terminal signal peptide. Underlined are the restriction enzyme sites for XmaI (forward primer) and XhoI (reverse primer) to enable insertion into the vector. The reverse primer also encodes His_6_-tag to provide an alternative protein purification method. For AliB-like ORF 2, forward primer 5′-TTTCCCGGGCGGGACAATCAGGTTCAGATAC-3′ and reverse primer 5′-AAACTCGAGTTAATGATGATGATGATGATGACCCCATTTGGTCTTAGCTTCTTC-3′ were used to amplify *aliB*-like ORF 2 without the 5′ portion encoding the predicted N-terminal signal peptide but with an XmaI site, an XhoI site and a His_6_-tag. The amplification products were ligated into expression plasmid pGEX 6p-1 which has an ampicillin resistance cassette. The construct was used to transform the *E. coli* which harbour a ‘codon plasmid’ adapted for Gram-positive bacteria which contains a chloramphenicol selection marker.

Expression of GST-*aliB*-like ORF1-His_6_ and GST-*aliB*-like ORF2-His_6_ was induced by isopropyl-β-d-thiogalacto-pyranoside (IPTG), final concentration 0.1 mM, shaking overnight at 20°C. Native AliB-like ORF 1 was isolated by binding the GST-tag to GSH-agarose beads (GE Healthcare) and native AliB-like ORF 2 was isolated by binding the His_6-_tag to nickel-nitrilotriacetic acid (Ni-NTA) agarose (Qiagen). The proteins were analysed by SDS-PAGE and shown to contain more than 95% purity of proteins of the expected sizes. The identity of the recombinant proteins was confirmed by peptide mass fingerprinting.

### 3.5. Identification of peptide ligands of AliB-like ORF 1 and ORF 2

Ligand-binding properties were investigated by incubating the recombinant proteins with 300 µl infant nasopharyngeal wash at 37°C on a turning wheel for 1 h to allow binding. Nasopharyngeal wash was obtained in the context of a larger study approved by the Institutional Ethical Board (Direktion Lehre und Forschung, Inselspital, University of Bern, 3010 Bern, Switzerland) and stored at 4°C until use. Written, informed consent was obtained.

The proteins (with ligands bound) were then recovered as described above using GSH-agarose beads to isolate AliB-like ORF 1 and Ni-NTA agarose to isolate AliB-like ORF 2. Bound ligands were released by denaturing the proteins with 8 M urea. As a negative control, the same procedure was performed in parallel with the exception that no IPTG was added to the bacteria. Therefore, specific induction of AliB-like ORF 1 and ORF 2 proteins was avoided in the negative control, leaving only expression of the *E. coli* proteins. Released peptides were isolated using 30 KDa filters then analysed by LC-MS. Peptide sequencing was performed on a linear trap quadrupole (LTQ) XL-Orbitrap mass spectrometer (Thermo Fisher Scientific, Bremen, Germany) with a Rheos Allegro nano flow system with AFM flow splitting (Flux Instruments, Reinach, Switzerland) and nano electrospray ion source operated at a voltage of 1.7 kV. Peptide separation was performed at least twice on a Magic C18 nano column (5 µm, 100 Å, 0.075 × 70 mm) at a flow rate of approximately 400 nl min^−1^, linear gradient (40 min) from 5 to 40% acetonitrile in water and 0.1% (v/v) formic acid. Data acquisition was in data-dependent mode on the top five peaks with mass-to-charge ratio (*m*/*z*) between 360 and 1400, exclusion 15 s. Survey full scan MS spectra were from 300 to 1800 *m/z*, resolution set at 60 000 (at 400 *m/z*). Fragmentation was by collision-induced dissociation with helium gas in the LTQ, and spectra were recorded at least once in the LTQ and once in the orbitrap with resolution 7500. Mascot generic files (mgf) were created by a pearl script using Hardklor software, from the *.RAW files [21]. MS/MS data were submitted to Phenyx (Geneva Bioinformatics) to search all bacteria or mammalian protein sequences in UniprotKB protein database (version 2010_12 as of 30 November 2010). Search parameters were; parent error tolerance 20 ppm, C-terminal cleavage after amino acids ACDEFGHIKLMNQRSTVWY, dynamic amino acid modifications of one Oxidation_M and one deamidation on Asn/Gln, minimal peptide *z*-score of 5, max *p*-value 0.01. The apparent false peptide identification rate was approximately 30% as estimated by a parallel search against the reversed database. The discovery of peptides specifically bound to the AliB-like ORFs was achieved by manual comparison of the negative with the positive control or using the software SIEVE (ThermoFisher Scientific).

### 3.6. Tryptophan binding assay

We used 3K Amicon Ultra centrifugal filters (Millipore, Carrigtwohill, Ireland) to exchange the elution buffer for the binding assay buffer. Tryptophan fluorescence spectroscopy assay was performed to measure binding of the recombinant proteins to the peptide ligands identified by mass spectroscopy, and to other peptides (PolyPeptide Group, Strasbourg, France), based on methods described previously [22,23]. The assay was performed by adding peptides dissolved in 10 mM NaH_2_PO_4_ to 0.5 μM GST-AliB-like ORF 1-His_6_ or GST-AliB-like ORF2-His_6_ in 1.5 ml 50 mM Tris–HCl pH 8.0 to a final concentration of 250 μM. Fluorescence was measured by LS-5B luminescence spectrometer (PerkinElmer, Schwerzenbach, Switzerland), connected to a graph printer, at excitation wavelength 295 nm (slit width 5 nm) and emission wavelength 330 nm (slit width 10 nm) while stirring continuously at 21°C.

### 3.7. Transformation assay

To compare transformation rates of 110.58 and its mutants, bacteria were grown to an OD_600nm_ of 0.15 in BHI containing 5% FCS. A total of 0.5 ml of the culture was transferred to 9.5 ml trypticase soy broth (TSB) competence medium pH 8.0 prewarmed to 30°C and incubated for 15 min. Competence-stimulating peptide 2 (CSP 2; EMRISRIILDFLFLRKK) was added to final concentration 100 ng ml^−1^ and the culture incubated for 15 min at 30°C. One microgram of chromosomal DNA from streptomycin resistant strain 104.37 was added and the culture incubated for 60 min at 30°C, then 90 min at 37°C. Serial dilutions in PBS were plated onto CSBA plates with and without 200 μg ml^−1^ streptomycin. After overnight incubation, the number of colonies was counted and the transformation rate calculated.

### 3.8. Competence gene expression

Competence development was measured using a modified version of the method of Caymaris *et al*. [24]. Stock cultures were grown at 37°C in TSB pH 8 to OD_600nm_ of 0.2 and frozen with 15% glycerol at −80°C. Thawed cultures were used to inoculate (1 : 10) C + Y medium containing luciferin (0.18 mg ml^−1^). Three hundred microlitres per well was added to a 96-well plate. To determine the synergistic effect of AliB-like ORF 1 ligand (SETTFGRDFN) on CSP 2-mediated competence induction, the bacteria were pre-treated for 30 min with AliB-like ORF 1 ligand (100 μg ml^−1^) before addition of 100 ng ml^−1^ CSP 2. To measure competence, we used *ssbB::luc* transcriptional fusion (kindly provided by M. Prudhomme and J. P. Claverys, Centre National de la Recherche Scientifique, Toulouse, France), which reports competence through light emission by luciferase [7]. Relative luminescence unit and OD values were recorded throughout incubation at 37°C (in a Varioskan Flash luminometer; Thermo Fisher Scientific, Zurich, Switzerland). Area under the curve (AUC) was calculated using GraphPad Prism 5.

### 3.9. Adherence assay

Adherence to an epithelial cell line was performed as described previously [14] with the following differences: Detroit 562 nasopharyngeal epithelial cells (ATCC CCL-138) were cultured on glass coverslips in a 24-well tissue culture plate and the number of bacteria adhered per Detroit cell was counted by microscopy following Giemsa staining. A total of 4 × 10^5^ cells/well in Minimum Essential Medium+Earle's salts (MEM) containing 10% FCS, 2 mM l-glutamine, 1.5 g l^−1^ sodium bicarbonate, 0.1 mM non-essential amino acids and 1 mM sodium pyruvate (Gibco, UK) were cultured until reaching complete confluence at 37°C in 5% CO_2_. *Streptococcus pneumoniae* strain 110.58 and its AliB-like ORF 1 and 2 deficient mutant grown in BHI broth containing 5% FCS to the logarithmic phase were washed in MEM then resuspended to give 10^7^ bacteria in 0.5 ml MEM, which was added per well of washed Detroit cells (the concentration of bacteria was confirmed by plating out appropriate dilutions of the inoculum). The bacteria were centrifuged onto the cells at 390*g* for 5 min at room temperature. After the 1 h incubation of the cells with the bacteria at 37°C and five washes with PBS, the cells were fixed by a 3 min incubation with 500 µl methanol, which was then removed. Four hundred microlitres of Giemsa stain (1 : 7 dilution of Giemsa in 0.007 M phosphate buffer, pH 6.8) was then added and incubated for 30 min and then removed. Following a 2 min wash with double distilled water, 500 µl 0.01% acetic acid was added and then immediately removed. This was repeated four times followed by a 2 min wash with double distilled water. When dry, the coverslip was mounted onto a microscope slide, viewed microscopically and photographed to allow counting of the number of bacteria adhered per cell.

### 3.10. Growth assays

To test the effect of the ORF 1 and ORF 2 ligand peptides on growth, pneumococci were streaked onto CSBA plates and cultured for 1 day at 37°C in a 5% CO_2_-enriched atmosphere. The resulting colonies were used to inoculate 5 ml BHI containing 5% FCS and cultured overnight at 37°C in a waterbath until OD_600nm_ was greater than 0.5. Two hundred microlitres of the overnight culture was added to 5 ml BHI containing 5% FCS with or without 1 mg ml^−1^ of the peptide ligand. After 4.5 h, serial dilutions were made and plated out on CSBA plates containing 500 µg ml^−1^ kanamycin. Colonies were counted the next day.

To test the effect of *E. coli* bacteria on pneumococcal growth, *E. coli* were streaked onto LB plates and *S. pneumoniae* strains were plated onto CSBA plates and cultured overnight at 37°C in a 5% CO_2_-enriched atmosphere. Overnight cultures of *S. pneumoniae* were prepared by inoculating 5 ml BHI with 5% FCS with colonies from the plate and by culturing the *E. coli* in LB broth with shaking. Two hundred microlitres of the overnight pneumococcal culture was then used to inoculate 5 ml BHI with 5% FCS plus either 200 µl of the *E. coli* overnight culture or 200 µl of LB broth. After 4.5 h, serial dilutions were made and plated out on CSBA plates containing 500 µg ml^−1^ kanamycin (except for control strain R6 which was plated on CSBA plates). After overnight incubation, the colonies were counted.

### 3.11. Nasopharyngeal colonization study

Female outbred MF1 mice were used. All protocols were approved by appropriate UK Home Office licensing authorities and by the University of Liverpool Ethical Committee. Mice were 8–10 weeks old when infected and weighed 30–35 g (Harlan, Bicester, UK). The mice were specific pathogen free but not germ free and therefore carrying their natural microflora. The mice were lightly anaesthetized with 2.5% (vol/vol) fluothane (AstraZeneca, Macclesfield, UK) over oxygen (1.5–2 l min^–1^), and 10 µl PBS containing 5 × 10^5^ cfu bacteria administered into the nostrils. The inoculum dose was confirmed by viable count after plating on blood agar plates following infection. At intervals following infection, groups of mice were killed by cervical dislocation, and the nasopharynx removed as previously described [25] and placed in 5 ml of sterile PBS, weighed and homogenized by an Ultra-Turrax hand-held homogenizer (IKA, Germany). Colony-forming units in homogenates were determined by serial dilution in sterile PBS and plating onto agar plates (Oxoid, Basingstoke, UK) with 5% (vol/vol) horse blood.

### 3.12. Genetic comparison

Genetic comparisons between species were made by performing BLAST searches using the NCBI website (http://www.ncbi.nlm.nih.gov/BLAST/).

### 3.13. Statistical analysis

Student *t*-tests were performed to obtain *p*-values.

## 4. Results

**Figure 1.**
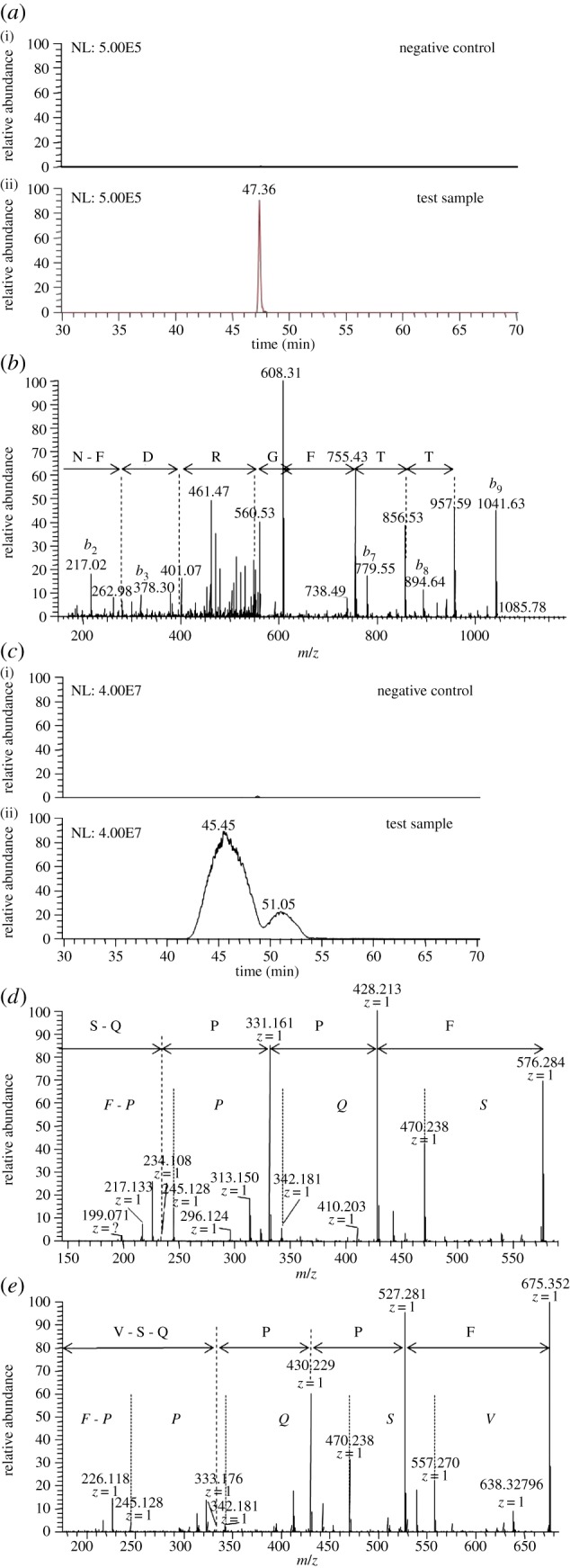
Identification of AliB-like ORF 1 ligand SETTFGRDFN and AliB-like ORF 2 ligands FPPQSV and FPPQS. (*a*) AliB-like ORF 1 ligand: extracted ion chromatogram of the doubly charged peptide ion with mass-to-charge ratio (*m*/*z*) of 587.26201 (retention time (RT) 47.36 min) with mass tolerance of ±1 ppm from the negative control (i) and positive sample (ii), intensity scale set at the same level. (*b*) Representative fragment mass spectrum acquired in the linear trap quadrupole (LTQ) iontrap and sequence interpretation by Phenyx software. Only the *y*-ion series (fragments appearing to extend from the carboxyl terminus) is annotated. (Individual peaks corresponding to the initial two amino acids S and E at the amino terminus are not visible in the *y*-ion series and are therefore not annotated but SE is the mass difference between the last detectable *y*-ion at 957.59 and the singly charged molecular ion of 1173.517.) (*c*) AliB-like ORF 2 ligands: extracted ion chromatogram of the singly charged peptide ions with mass-to-charge ratio (*m*/*z*) of 575.27934 (RT = 45.45 min, corresponding to sequence FPPQS) and 674.34844 (RT = 51.05 min, sequence FPPQSV) with mass tolerance of ±2 ppm from the negative control (i) and positive sample (ii), intensity scale set at the same level. A representative fragment mass spectrum acquired in Fourier transformation (FT) mode, resolution of 7500, of peptides 575 (*d*) and 674 (*e*) is shown. Manual interpretation of the fragment peaks is shown using italic letters for the *b*-ion (fragments appearing to extend from the amino terminus) and non-italic letters for the *y*-ion series. Confirmation of correct interpretation was achieved with fragment spectra of synthetic peptides (electronic supplementary material, figure S3).

To find the ligands of *S. pneumoniae* proteins AliB-like ORF 1 and ORF 2, we expressed recombinant proteins and incubated them in human nasopharyngeal washings and then released bound peptide with urea. LC-MS analysis identified a single peptide ligand for AliB-like ORF 1 with the sequence: SETTFGRDFN (figure 1*a*,*b*). For AliB-like ORF 2, two peptide ligands were identified: FPPQSV and FPPQS (figure 1*c*–*e* and electronic supplementary material, figure S3). BLAST analysis indicated that AliB-like ORF 1 ligand is in the 50S ribosomal subunit protein L4 of Enterobacteriaceae, including *Salmonella enterica, E. coli, Serratia symbiotica* and *Klebsiella pneumoniae*. The sequence of the AliB-like ORF 2 ligand FPPQSV also has multiple bacterial matches including ribosome-associated GTPase EngA of the human commensal *Prevotella salivae* and sulfate adenylyltransferase of *Prevotella tannerae*.

**Figure 2.**
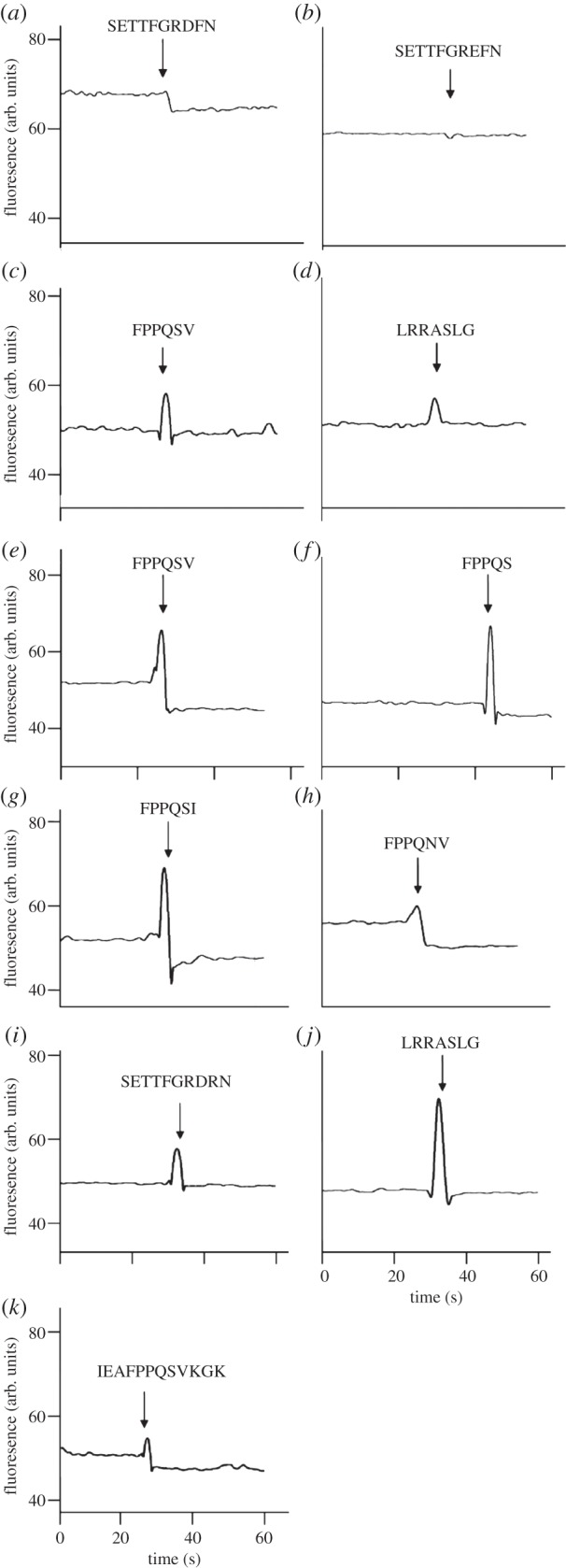
Binding of recombinant AliB-like ORFs to their ligands. Binding of recombinant AliB-like ORF 1 (*a*–*d*) and AliB-like ORF 2 (*e*–*k*) to synthetic peptides was determined by measuring changes in intrinsic protein fluorescence using tryptophan fluorescence. A drop in fluorescence indicates binding. Peptide sequences are indicated above the arrows marking the moment of their addition to the protein.

Tryptophan fluorescence binding assays confirmed that the ligand of AliB-like ORF 1 is SETTFGRDFN (figure 2*a*). (Testing of a longer peptide containing the AliB-like ORF 1 ligand sequence was prevented by its insolubility.) Binding was specific as a peptide with one amino acid difference (SETTFGREFN), the equivalent sequence in *Haemophilus influenzae,* a human pathogen which colonizes the nasopharynx, did not bind (figure 2*b*). Also, AliB-like ORF 2 ligand FPPQSV (figure 2*c*) and the putative ligand for real-AliB (LRRASLG) (figure 2*d*) did not bind AliB-like ORF 1 protein. The tryptophan fluorescence assay also confirmed that the ligand of AliB-like ORF 2 is FPPQSV (figure 2*e*) with a positive, but weaker, signal with FPPQS (figure 2*f*). AliB-like ORF 2 was more promiscuous than AliB-like ORF 1 as AliB-like ORF 2 protein also bound FPPQSI (*P. tannerae*, putative ABC transporter) (figure 2*g*) and FPPQNV (human Deltex 1) (figure 2*h*) though it did not bind the completely unrelated peptides of the AliB-like ORF 1 ligand (figure 2*i*) or the ligand for real-AliB (LRRASLG) (figure 2*j*). Binding of AliB-like ORF 2 protein was also observed with peptide IEAFPPQSVKGK (figure 2*k*) representing AliB-like ORF 2 ligand (underlined) flanked by three amino acids either side that are found adjacent to the ligand sequence in *P. salivae* GTPase EngA.

**Figure 3.**
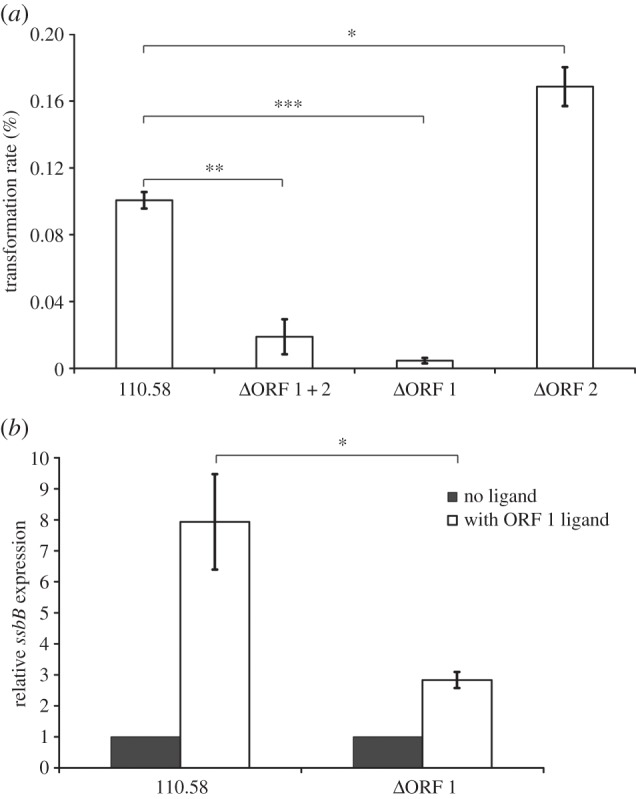
Competence gene expression and transformation. (*a*) Percentage transformation rates of strain 110.58 and its mutants ΔORF 1 + 2, ΔORF 1 and ΔORF 2, showing the mean of three independent experiments. Error bars show s.e.m. *p = 0.0058, **p = 0.0022, ***p < 0.0001. (*b*) The synergistic effect of pre-incubation with 100 µg ml^−1^ AliB-like ORF 1 ligand (SETTFGRDFN) on CSP 2-mediated competence induction was determined by measuring the expression of late competence gene *ssbB* using a transcriptional fusion of the *ssbB* promoter region with the luciferase gene, displayed as AUC of the luminescence signal. Results represent three experiments and show s.e.m. For each strain, the expression of *ssbB* in the presence of ORF 1 ligand is presented as a fold difference compared with the expression value of that strain in the absence of the ORF 1 ligand. *p = 0.0311.

To enable functional analyses of the AliB-like ORFs, we made mutants where one or both ORFs were disrupted (see the electronic supplementary material, figure S1 for genetic structures). Since AliB-like ORF 1 bound a peptide fragment that is found in Enterobacteriaceae, which are not part of the normal nasopharyngeal microbiota, we speculated that such binding may elicit a stress response. *Streptococcus pneumoniae* are naturally competent for genetic transformation and being able to take up DNA in a hostile environment may confer a survival advantage. Transformation rate was therefore compared in the wild-type and mutant strains. Wild-type strain 110.58 had a transformation rate 5.3-fold higher than that of the mutant lacking both ORFs, and 20.2-fold higher than that of the mutant lacking ORF 1 only (figure 3*a*). The mutant lacking only ORF 2 had a higher transformation rate than the wild-type strain. We confirmed the role of AliB-like ORF 1 in competence by investigating expression of late competence gene *ssbB* in wild-type strain 110.58 and its ΔORF 1 mutant (figure 3*b*). We observed no increase in spontaneous competence induction by incubating with AliB-like ORF 1 ligand SETTFGRDFN without adding competence-stimulating peptide (CSP) 2, the peptide released by *S. pneumoniae* that triggers the cascade leading to competence for genetic transformation (C Hauser 2012, unpublished data). However, pre-incubation with AliB-like ORF 1 ligand increased the competence response to CSP 2 to a significantly greater extent in the wild-type strain 110.58 than in the ΔORF 1 mutant. (The increase in relative *ssbB* expression in the ΔORF 1 mutant with ORF 1 ligand is equivalent to that observed with a negative control peptide of unrelated sequence, data not shown.)

**Figure 4.**
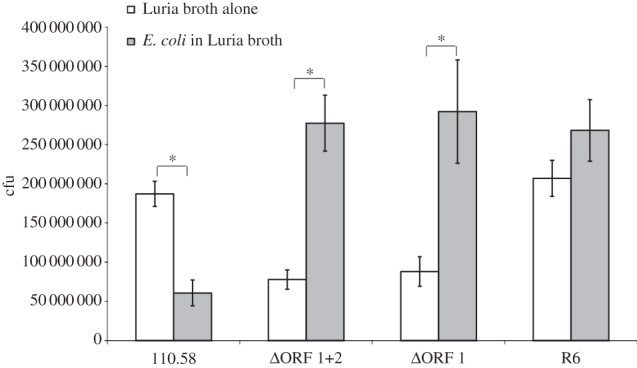
Effect of incubation with *E. coli* on pneumococcal growth. The wild-type strain 110.58 and its mutants, as well as the control laboratory strain R6, were assessed for growth in the presence and absence of *E. coli* bacteria by quantifying colony forming units (cfu). Data represent the mean of three independent experiments. Error bars show s.e.m. *p < 0.05.

The effect of the ligands on growth of the wild-type strain and its mutants was also tested by exposing the bacteria to the free peptides, but no effect was observed (electronic supplementary material, figure S4). We also tested whether ORF 1 ligand had any effect on expression of *aliB*-like ORF 1 or 2 at different stages of the growth curve, but found it had none (electronic supplementary material, figure S5). We therefore went on to test whether culture with *E. coli* bacteria (in which the ORF 1 ligand is found within the 50S ribosomal subunit protein L4) affected growth of the pneumococci. Figure 4 shows that *E. coli* did indeed have a significant inhibitory effect on growth which depended on the presence of AliB-like ORF 1. In the mutants lacking AliB-like ORF 1 *E. coli* increased growth, an effect which was also seen, to a lesser extent, in the laboratory pneumococci strain R6 which also lacks AliB-like ORF 1.

**Figure 5.**
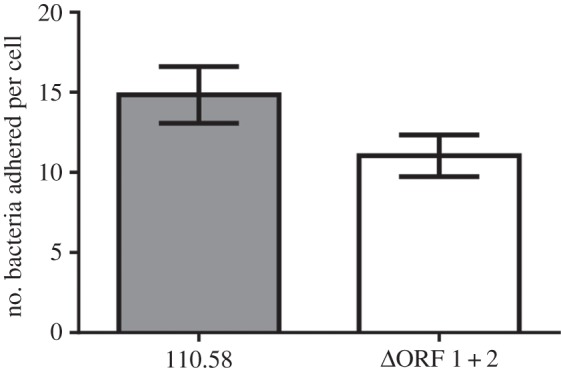
Adherence to Detroit 562 nasopharyngeal epithelial cells. The number of adhered bacteria per Detroit cell was counted by microscopy following Giemsa staining. Data represent the mean of four independent experiments. Error bars show s.e.m.

**Figure 6.**
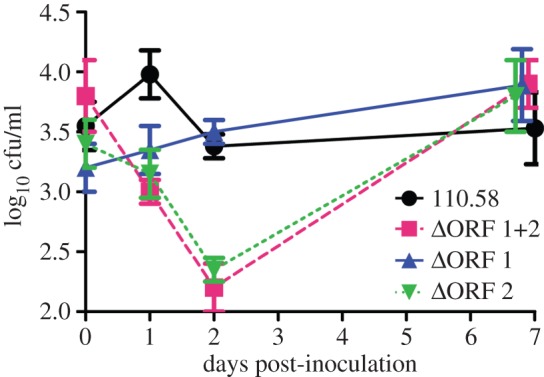
Colonization of the nasopharynx of female MF1 mice. Quantification of bacterial load in the nasopharynx at different timepoints post-inoculation was determined by plating out dilutions of nasopharyngeal tissue homogenate and counting the number of colony-forming units for the wild-type strain 110.58 (solid lines with circles), its mutant lacking the *aliB*-like ORFs (ΔORF 1 ± 2) (dashed lines with squares), ΔORF 1 (solid line with triangles) and ΔORF 2 (dotted lines with inverted triangles). All data derive from five mice per timepoint per strain tested. Data are expressed as mean log_10_ cfu/ml nasopharyngeal tissue ± s.e.m.

AliB-like ORF 2 protein bound ligands that matched peptides of the nasopharyngeal commensal species *Prevotella*, a frequent component of the human nasopharyngeal microbiota. When we compared adherence of wild-type strain 110.58 and mutant ΔORF 1 + 2 to Detroit 562 human nasopharyngeal epithelial cells we found a slightly (non-significant, *p* = 0.13) greater adherence for the wild-type strain (figure 5), and so we speculated that the phenotype related to ORF 2 may be enhanced colonization *in vivo*. We measured colonization of the mouse nasopharynx for wild-type strain 110.58 and its ORF 1 and ORF 2 mutants. At 20 min post-inoculation, there was no difference in nasopharyngeal colonization between any of the strains; however at 24 and 48 h, there was significantly (*p* < 0.05) less colonization by the ΔORF 1 + 2 and ΔORF 2 mutants than the wild-type strain 110.58 (figure 6). By day 7, the differences had disappeared. There was no significant difference in colonization between the wild-type strain and ΔORF 1.

## 5. Discussion

We aimed to determine whether there is evidence for interspecies communication between bacteria that live in the human nasopharynx. We found that *S. pneumoniae* are able to recognize and respond to peptides matching other bacterial species.

We identified short peptide fragments that, via their receptors AliB-like ORF 1 and ORF 2, may mediate responses to the microbial environment. Peptide fragments that indicated the presence of Enterobacteriaceae facilitated an upregulation of competence for genetic transformation. Secreted proteins of *E. coli* include ribosomal associated proteins of the 50S ribosomal subunit [26]. Therefore, such proteins may have a role in communication as well as ribosome function and assembly [27]. Prudhomme *et al.* [7] showed that low concentrations of antibiotics induce a stress response in *S. pneumoniae*, leading to an upregulation of spontaneous competence, and they compared this to the SOS response in *E. coli* induced by the DNA-damaging agent mitomycin C and by fluoroquinolones. Here, we propose that when the peptide fragment of Enterobacteriaceae binds to AliB-like ORF 1 protein it triggers a similar stress response, which manifests itself as a synergistic increase in competence. This would allow the pneumococci to take up DNA that could give it an advantage in terms of adapting to the stressful environment. A difference between our finding and that of Prudhomme *et al.* is that they found that antibiotics were sufficient alone to stimulate the competence response. By contrast, to observe the effect of peptide on competence, we needed to supply exogenous CSP. This indicates that in nature another signal is required to induce the stress response leading to competence. *Streptococcus pneumoniae* spontaneously release CSP and so maybe local concentrations of CSP and peptide would be enough to start the competence cascade. Alternatively, binding of other signals to their receptors could act synergistically with the binding of peptide to AliB-like ORF 1 protein to initiate the stress response.

We did not find an effect of free ORF 1 ligand on growth but when *E. coli* (which produce the ORF 1 ligand) were cultured together with the pneumococci they inhibited the pneumococcal growth, and this was dependent on the presence of AliB-like ORF 1. We speculate that in the nasopharynx, detection of Enterobacteriaceae may trigger *S. pneumoniae* that have AliB-like ORF 1 to stop growing and become competent.

We propose that when *S. pneumoniae* bind peptides from *Prevotella* species via AliB-like ORF 2 this triggers *S. pneumoniae* to promote colonization of the mucosa and may decrease competence for genetic transformation. This is supported by the fact that in our mouse nasopharyngeal colonization experiments, knocking out AliB-like ORF 2 reduced colonization at the early timepoints. Although we did not find a significant difference in adherence to epithelial cells *in vitro*, the adherence of the wild-type strain was slightly greater than that of the mutant lacking the AliB-like ORFs and so we speculate that the difference in early colonization efficiency *in vivo* could be related to an advantage in adherence for bacteria possessing AliB-like ORF 2*.* Alternatively, or in addition, another possibility is that signalling via AliB-like ORF 2 might stimulate growth at the early stages of colonization. The results indicate some flexibility in the peptide length that AliB-like ORF 2 protein can bind as long as the ligand sequence is present. This would be relevant if *Prevotella* releases a mixture of peptides of differing lengths. Bacteria with the ability to sense a favourable environment with normal commensal species present might be well adapted to a commensal lifestyle.

An indication that AliB-like ORF 1 and ORF 2 may have a role wider than that shown here in non-encapsulated *S. pneumoniae* is that these genes are also found in strains of the commensal streptococci *S. oralis*, *S. mitis* and *S. pseudopneumoniae* [28–30]. The entire *aliB*-like ORF 2 is also found within the capsule operon of *S. pneumoniae* serotypes 25a, 25f and 38 and an *aliB*-like ORF 2 fragment in 66 other serotypes [31]. Although AliB-like ORF 1 and ORF 2 are predominantly found in non-encapsulated *S. pneumoniae* and in commensals, they are homologues of AmiA, AliA and AliB of the Ami-AliA/AliB permease, found in all *S. pneumoniae* strains [16]. We therefore speculate that AmiA, AliA and AliB may also be involved in interspecies bacterial communication, but this remains to be tested.

In this study, we found that AliB-like ORF 1 and ORF 2 proteins of *S. pneumoniae* bacteria bind short peptides matching sequences found in other bacterial species. Binding results in phenotypic changes indicating adaptation to the microbial community. AliB-like ORF 1 protein binds a peptide found in Enterobacteriaceae, bacteria only expected to be present in an unhealthy nasopharynx, indicating a stressful environment and triggering an increase in competence for genetic transformation. AliB-like ORF 2 protein binds a peptide found in *Prevotella* which might indicate a normal nasopharyngeal microbial community, triggering colonization.

We therefore propose that sensing of short peptides is a newly described mechanism which could be employed by bacteria to sense neighbouring species in the microbiota and adapt accordingly. Manipulation of this communication could be a new target in the control of bacterial colonization and infections.

## Supplementary Material

Figure S1

## Supplementary Material

Figure S2

## Supplementary Material

Figure S3

## Supplementary Material

Figure S4

## Supplementary Material

Figure S5

## References

[1] Ryan R Dow J 2008 Diffusible signals and interspecies communication in bacteria. Microbiology 154, 1845–1858. (doi:10.1099/mic.0.2008/017871-0) 18599814 10.1099/mic.0.2008/017871-0

[2] Schauder S Bassler B 2001 The languages of bacteria. Genes Dev. 15, 1468–1480. (doi:10.1101/gad.899601) 11410527 10.1101/gad.899601

[3] Lam H Oh D-C Cava F Takacs C Clardy J Pedro Md Waldor M 2009 d-Amino acids govern stationary phase cell wall remodeling in bacteria. Science 325, 1552–1555. (doi:10.1126/science.1178123) 19762646 10.1126/science.1178123 PMC2759711

[4] Kolodkin-Gal I Romero D Cao S Clardy J Kolter R Losick R 2010 d-Amino acids trigger biofilm disassembly. Science 328, 627–629. (doi:10.1126/science.1188628) 20431016 10.1126/science.1188628 PMC2921573

[5] Cava F Pedro Md Lam H Davis B Waldor M 2011 Distinct pathways for modification of the bacterial cell wall by non-canonical d-amino acids. The EMBO J. 30, 3442–3453. (doi:10.1038/emboj.2011.246) 21792174 10.1038/emboj.2011.246 PMC3160665

[6] Davies J Spiegelman G Yim G 2006 The world of subinhibitory antibiotic concentrations. Curr. Opin. Microbiol. 9, 445–453. (doi:10.1016/j.mib.2006.08.006) 16942902 10.1016/j.mib.2006.08.006

[7] Prudhomme M Attaiech L Sanchez G Martin B Claverys J 2006 Antibiotic stress induces genetic transformability in the human pathogen *Streptococcus pneumoniae*. Science 313, 89–92. (doi:10.1126/science.1127912) 16825569 10.1126/science.1127912

[8] Claverys J Prudhomme M Mortier-Barriere I Martin B 2000 Adaptation to the environment: *Streptococcus pneumoniae*, a paradigm for recombination-mediated genetic plasticity? Mol. Microbiol. 35, 251–259. (doi:10.1046/j.1365-2958.2000.01718.x) 10652087 10.1046/j.1365-2958.2000.01718.x

[9] Hilty M 2010 Disordered microbial communities in asthmatic airways. PLoS ONE 5, e8578 (doi:10.1371/journal.pone.0008578) 20052417 10.1371/journal.pone.0008578 PMC2798952

[10] Hilty M Qi W Brugger S Frei L Agyeman P Frey P Aebi S Muhlemann K 2012 Nasopharyngeal microbiota in infants with acute otitis media. J. Infect Dis. 205, 1048–1055. (doi:10.1093/infdis/jis024) 22351941 10.1093/infdis/jis024 PMC7107284

[11] Carvalho M Steigerwalt A Thompson T Jackson D Facklam R 2003 Confirmation of nontypeable *Streptococcus pneumoniae*-like organisms isolated from outbreaks of epidemic conjunctivitis as *Streptococcus pneumoniae*. J. Clin. Microbiol. 41, 4415–4417. (doi:10.1128/JCM.41.9.4415-4417.2003) 12958280 10.1128/JCM.41.9.4415-4417.2003 PMC193841

[12] Finland M Barnes M 1977 Changes in occurrence of capsular serotypes of *Streptococcus pneumoniae* at Boston City Hospital during selected years between 1935 and 1974. J. Clin. Microbiol. 5, 154–166. 14971 10.1128/jcm.5.2.154-166.1977 PMC274557

[13] van der Windt D Bootsma HJ Burghout P van der Gaast-de Jongh CE Hermans PWM van der Flier M 2012 Nonencapsulated *Streptococcus pneumoniae* resists extracellular human neutrophil elastase- and cathepsin G-mediated killing. FEMS Immunol. Med. Microbiol. 66, 445–448. (doi:10.1111/j.1574-695X.2012.01028.x) 22943431 10.1111/j.1574-695X.2012.01028.x

[14] Hathaway L Meier PS Battig P Aebi S Muhlemann K 2004 A homologue of *aliB* is found in the capsule region of nonencapsulated *Streptococcus pneumoniae*. J. Bacteriol. 186, 3721–3729. (doi:10.1128/JB.186.12.3721-3729.2004) 15175285 10.1128/JB.186.12.3721-3729.2004 PMC419944

[15] Polissi A Pontiggia A Feger G Altieri M Mottl H Ferrari L Simon D 1998 Large-scale identification of virulence genes from *Streptococcus pneumoniae*. Infect. Immun. 66, 5620–5629. 9826334 10.1128/iai.66.12.5620-5629.1998 PMC108710

[16] Claverys J Grossiord B Alloing G 2000 Is the Ami-AliA/B oligopeptide permease of *Streptococcus pneumoniae* involved in sensing environmental conditions? Res. Microbiol. 151, 457–463. (doi:10.1016/S0923-2508(00)00169-8) 10961459 10.1016/s0923-2508(00)00169-8

[17] Hathaway L Brugger S Martynova A Aebi S Muhlemann K 2007 Use of the Agilent 2100 bioanalyzer for rapid and reproducible molecular typing of *Streptococcus pneumoniae*. J. Clin. Microbiol. 45, 803–809. (doi:10.1128/JCM.02169-06) 17202282 10.1128/JCM.02169-06 PMC1829109

[18] Hanahan D 1983 Studies on transformation of *Escherichia coli* with plasmids. J. Mol. Biol. 166, 557–580. (doi:10.1016/S0022-2836(83)80284-8) 6345791 10.1016/s0022-2836(83)80284-8

[19] Lacks S Lopez P Greenberg B Espinosa M 1986 Identification and analysis of genes for tetracycline resistance and replication functions in the broad-host-range plasmid pLS1. J. Mol. Biol. 192, 753–765. (doi:10.1016/0022-2836(86)90026-4) 2438417 10.1016/0022-2836(86)90026-4

[20] Hathaway L Battig P Muhlemann K 2007 *In vitro* expression of the first capsule gene of *Streptococcus pneumoniae*, *cpsA*, is associated with serotype-specific colonization prevalence and invasiveness. Microbiology 153, 2465–2471. (doi:10.1099/mic.0.2006/005066-0) 17660411 10.1099/mic.0.2006/005066-0

[21] Hoopmann M Finney G MacCoss M 2007 High-speed data reduction, feature detection, and ms/ms spectrum quality assessment of shotgun proteomics data sets using high-resolution mass spectrometry. Anal. Chem. 79, 5620–5632. (doi:10.1021/ac0700833) 17580982 10.1021/ac0700833 PMC2556510

[22] Thomas G Southworth T Leon-Kempis M Leech A Kelly D 2006 Novel ligands for the extracellular solute receptors of two bacterial TRAP transporters. Microbiology 152, 187–198. (doi:10.1099/mic.0.28334-0) 16385129 10.1099/mic.0.28334-0

[23] Basavanna S Khandavilli S Yuste J Cohen J Hosie A Webb A Thomas G Brown J 2009 Screening of *Streptococcus pneumoniae* ABC transporter mutants demonstrates that LivJHMGF, a branched-chain amino acid ABC transporter, is necessary for disease pathogenesis. Infect. Immun. 77, 3412–3423. (doi:10.1128/IAI.01543-08) 19470745 10.1128/IAI.01543-08 PMC2715661

[24] Caymaris S Bootsma H Martin B Hermans P Prudhomme M Claverys J 2010 The global nutritional regulator CodY is an essential protein in the human pathogen *Streptococcus pneumoniae*. Mol. Microbiol. 78, 344–360. (doi:10.1111/j.1365-2958.2010.07339.x) 20979332 10.1111/j.1365-2958.2010.07339.x

[25] Kadioglu A Taylor S Iannelli F Pozzi G Mitchell T Andrew P 2002 Upper and lower respiratory tract infection by *Streptococcus pneumoniae* is affected by pneumolysin deficiency and differences in capsule type. Infect. Immun. 70, 2886–2890. (doi:10.1128/IAI.70.6.2886-2890.2002) 12010976 10.1128/IAI.70.6.2886-2890.2002 PMC128015

[26] Lee E-Y 2007 Global proteomic profiling of native outer membrane vesicles derived from *Escherichia coli*. Proteomics 7, 3143–3153. (doi:10.1002/pmic.200700196) 17787032 10.1002/pmic.200700196

[27] Zaman S Fitzpatrick M Lindahl L Zengel J 2007 Novel mutations in ribosomal proteins L4 and L22 that confer erythromycin resistance in *Escherichia coli*. Mol. Microbiol. 66, 1039–1050. (doi:10.1111/j.1365-2958.2007.05975.x) 17956547 10.1111/j.1365-2958.2007.05975.x PMC2229831

[28] Reichmann P 2011 Genome of *Streptococcus oralis* strain Uo5. J. Bact. 193, 2888–2889. (doi:10.1128/JB.00321-11) 21460080 10.1128/JB.00321-11 PMC3133139

[29] Denapaite D 2010 The genome of *Streptococcus mitis* B6—what is a commensal? PLoS ONE 5, e9426 (doi:10.1371/journal.pone.0009426) 20195536 10.1371/journal.pone.0009426 PMC2828477

[30] Shahinas D 2011 Whole-genome sequence of *Streptococcus pseudopneumoniae* isolate IS7493. J. Bact. 193, 6102–6103. (doi:10.1128/JB.06075-11) 21994930 10.1128/JB.06075-11 PMC3194916

[31] Bentley S 2006 Genetic analysis of the capsular biosynthetic locus from all 90 pneumococcal serotypes. PLoS Genet. 2, e31 (doi:10.1371/journal.pgen.0020031) 16532061 10.1371/journal.pgen.0020031 PMC1391919

