# Supplementary material for: Streptococcus pneumoniae detects and responds to foreign bacterial peptide fragments in its environment
Source: Open Biol. 2014 Apr 9;4(4):130224. doi: 10.1098/rsob.130224 (PMC4043112; doi:10.1098/rsob.130224)
Supplement: Figure S1 [file rsob130224supp1.pdf]

Supplementary information

Figure S1

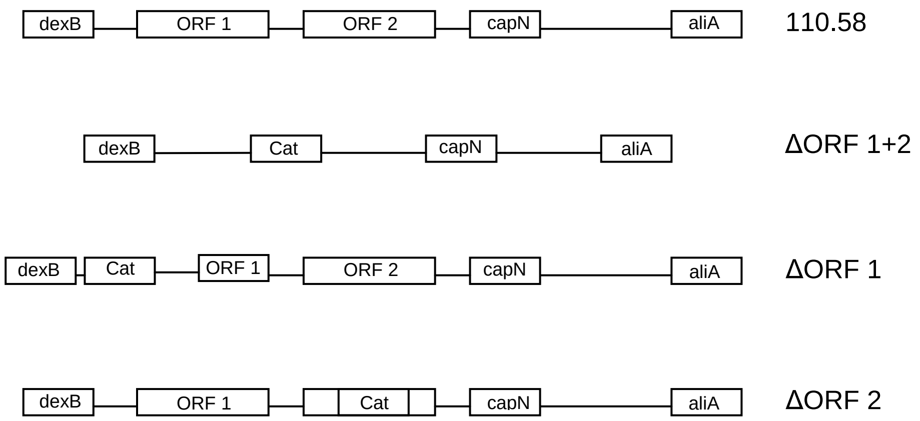

Figure S1. Diagram (not to scale) showing genetic structure of the capsule region of mutants made of the clinical isolate 110.58 which has *aliB-like* ORF 1 and ORF 2. Cat = chloramphenicol resistance gene, Spc = spectinomycin resistance gene.
