# Supplementary material for: Streptococcus pneumoniae detects and responds to foreign bacterial peptide fragments in its environment
Source: Open Biol. 2014 Apr 9;4(4):130224. doi: 10.1098/rsob.130224 (PMC4043112; doi:10.1098/rsob.130224)
Supplement: Figure S2 [file rsob130224supp2.pdf]

Figure S2

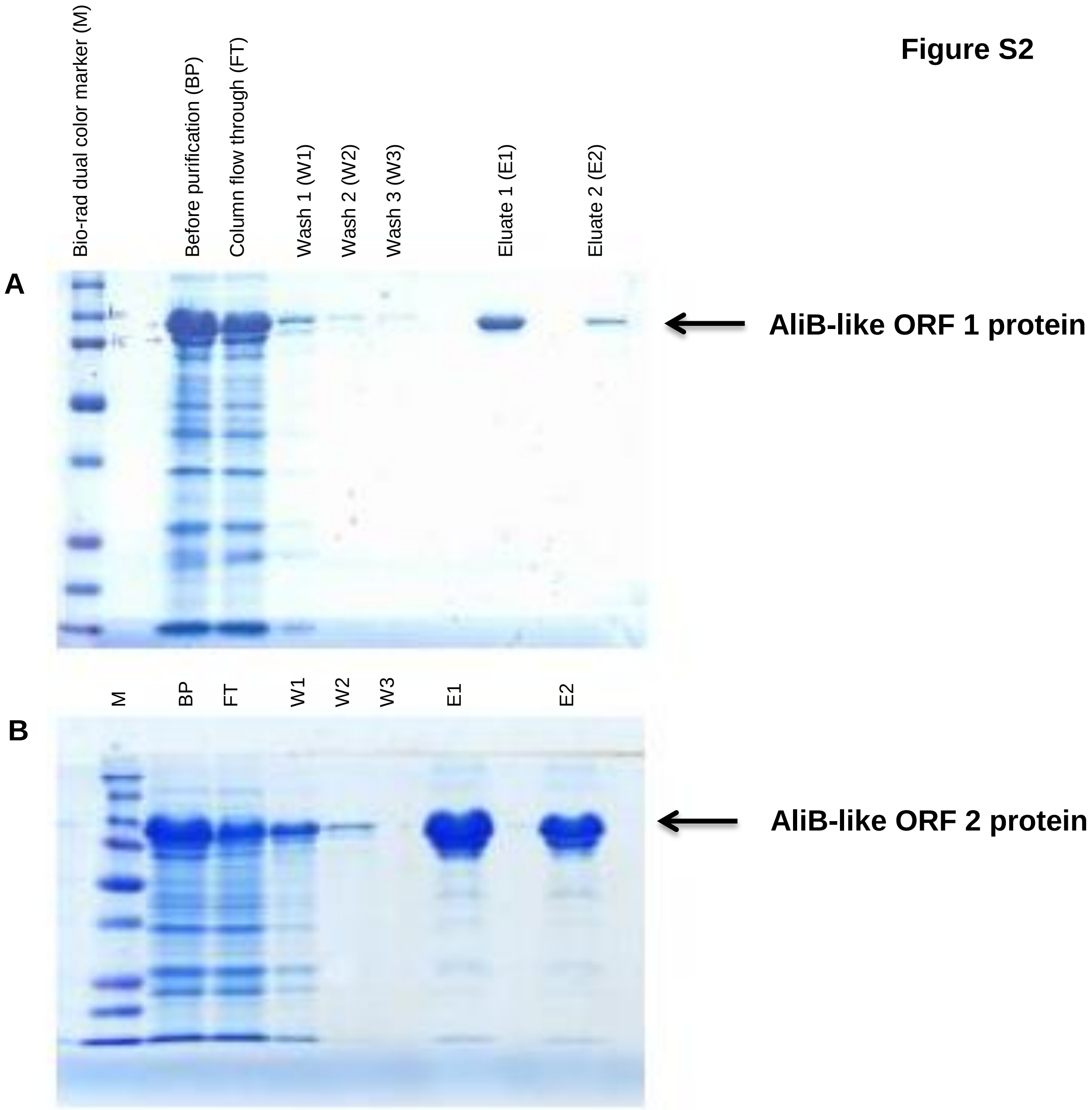

Figure S2. SDS-PAGE of proteins AliB-like ORF 1 (A) and AliB-like ORF 2 (B) before and after purification. The protocol for expression was based on the Qiagen QIAexpressionist kit as follows: To express the proteins the bacteria were streaked out on LB plates containing 100 µg/ml ampicillin and 30 µg/ml chloramphenicol and incubated overnight at 37ºC and the colonies used to inoculate LB broth containing 100 µg/ml ampicillin and 30 µg/ml chloramphenicol. After growth at 37ºC with shaking until OD_{600nm} = 0.4 expression was induced by adding IPTG to 0.1mM followed by overnight culture with shaking at 20ºC. The bacteria were recovered by centrifugation and the pellet resuspended in lysis buffer (50 mM NaH_2PO_4,300 mM NaCl,10 mM imidazole, pH adjusted to 8 using NaOH). Lysozyme was added to give 1 mg/ml, followed by incubation on ice for 30 minutes and then sonication 6 times for 10 seconds with 10 seconds cooling period between each sonication, maintaining on ice during sonication. The supernatant containing the soluble protein was recovered by centrifugation 4ºC, 10 000g for 20 min. Purification of AliB-like ORF 1 protein was by its N-terminal GST tag and purification of AliB-like ORF 2 proteins was by its C-terminal His_6 tag as described in the main text.
