# Supplementary material for: Streptococcus pneumoniae detects and responds to foreign bacterial peptide fragments in its environment
Source: Open Biol. 2014 Apr 9;4(4):130224. doi: 10.1098/rsob.130224 (PMC4043112; doi:10.1098/rsob.130224)
Supplement: Figure S3 [file rsob130224supp3.pdf]

Supplementary information

Figure S3

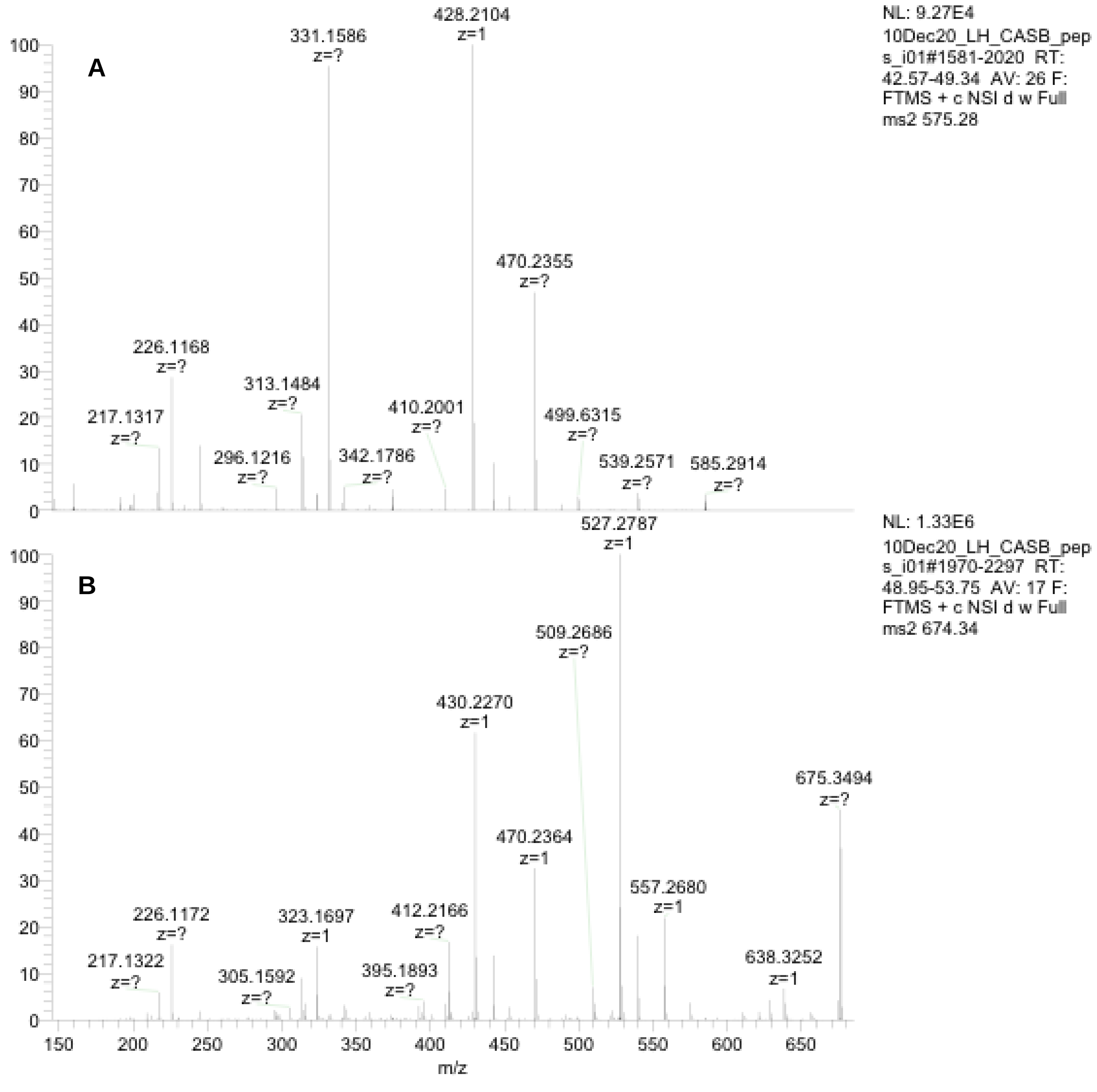

Figure S3. Fragment spectra of synthetic peptides (A) FPPQS and (B) FPPQSV.
