# Supplementary material for: Streptococcus pneumoniae detects and responds to foreign bacterial peptide fragments in its environment
Source: Open Biol. 2014 Apr 9;4(4):130224. doi: 10.1098/rsob.130224 (PMC4043112; doi:10.1098/rsob.130224)
Supplement: Figure S4 [file rsob130224supp4.pdf]

Figure S4

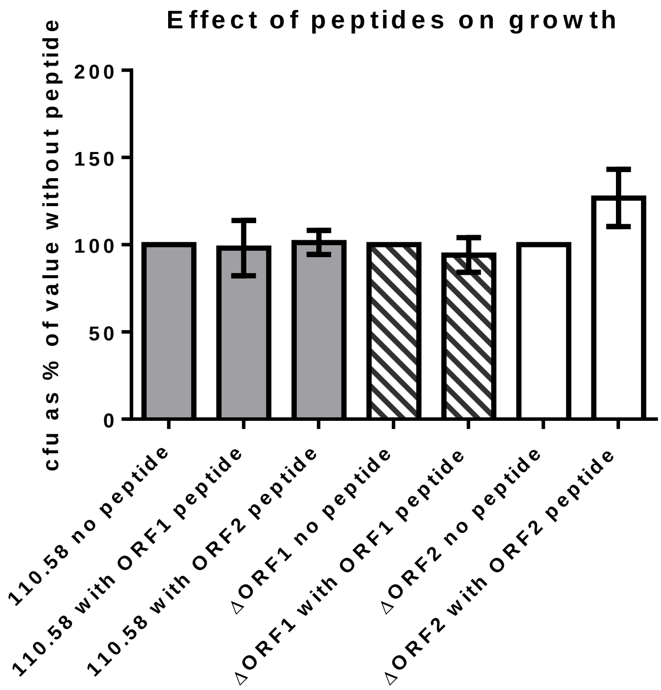

Figure S4. The effect of AliB-like ORF 1 and ORF 2 ligands on growth. No significant effect on the number of cfus was observed with either peptide on the wildtype strain 110.58 or its mutants lacking *aliB-like* ORF 1 or ORF 2.
