# Supplementary material for: Streptococcus pneumoniae detects and responds to foreign bacterial peptide fragments in its environment
Source: Open Biol. 2014 Apr 9;4(4):130224. doi: 10.1098/rsob.130224 (PMC4043112; doi:10.1098/rsob.130224)
Supplement: Figure S5 [file rsob130224supp5.pdf]

Supplementary Figure S5

A

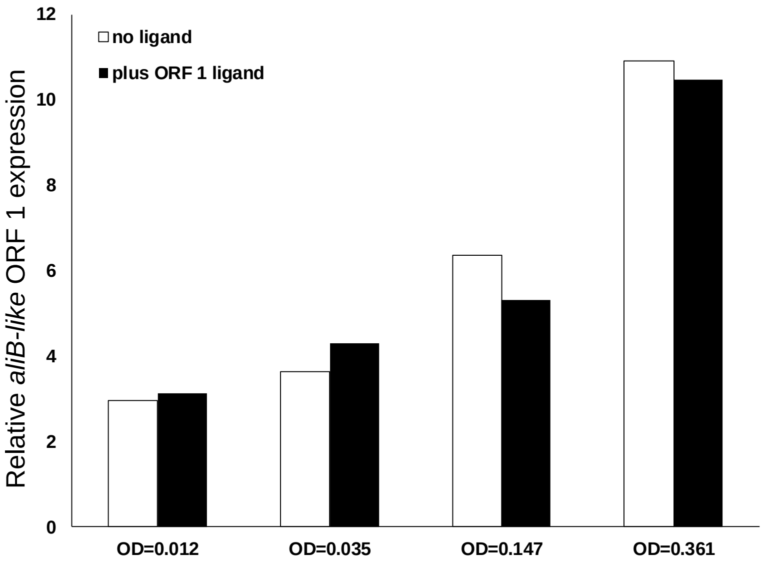

B

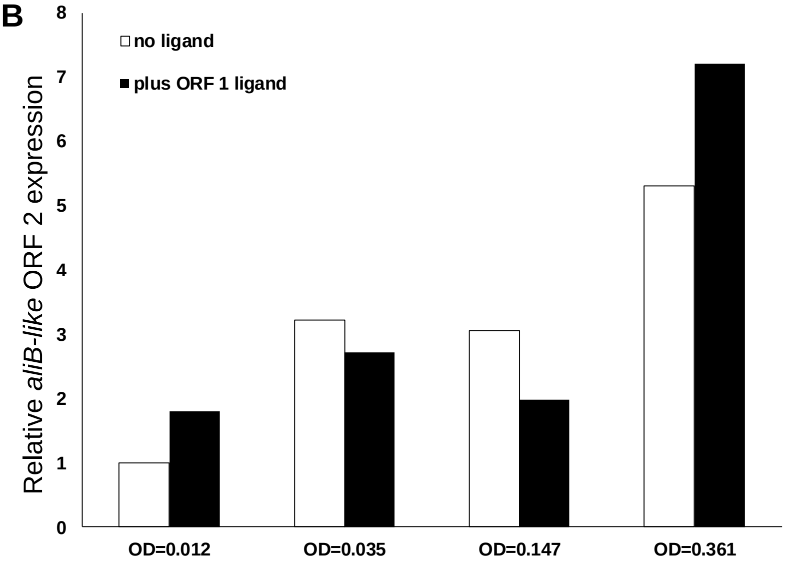

Figure S5. Real-time RT-PCR results of wildtype strain 110.58 at different OD_{600nm}, shown on the x axis, showing no effect of ORF 1 ligand on either *aliB-like* ORF 1 expression (A) or *aliB-like* ORF 2 expression (B).
